# The protective effect of alcohol consumption on the incidence of cardiovascular diseases: is it real? A systematic review and meta-analysis of studies conducted in community settings

**DOI:** 10.1186/s12889-019-7820-z

**Published:** 2020-01-21

**Authors:** Seok-Joon Yoon, Jin-Gyu Jung, Sami Lee, Jong-Sung Kim, Soon-ki Ahn, Ein-Soon Shin, Ji-Eun Jang, Sang-Hyun Lim

**Affiliations:** 1 Department of Family Medicine, Chungnam National University Hospital, 282, Munhwa-ro, Jung-gu, Daejeon, Republic of Korea; 2 Public Health and Medical Services Office, Chungnam National University Hospital, Daejeon, Republic of Korea; 3 KAMS Research Center, Research Agency for Clinical Practice Guidelines, Korean Academy of Medical Sciences (KAMS), Seoul, Republic of Korea; 4 Division of Cardiology, Department of Internal Medicine, The Catholic University of Korea, Seoul, Republic of Korea

**Keywords:** Alcohol drinking, Cardiovascular diseases, Residence characteristics, Comorbidity, Meta-analysis, Health promotion

## Abstract

**Background:**

This study investigated the dose-response relationship between alcohol consumption and CVD incidence, conducting a meta-analysis of studies focusing on residents from local communities. Further, we examined whether light to moderate alcohol consumption had a protective effect on CVD incidence through a sub-group analysis.

**Methods:**

This study conducted a meta-analysis of the relationship between alcohol consumption and CVD incidence, selecting journals published up to December 2017. The alcohol consumption level was classified into non-consumers, light (0.01–10.0 g/day), light to moderate (10.1–20.0 g/day), moderate (20.1–40.0 g/day), moderate to high (40.1–60.0 g/day), and high (> 60.0 g/day) groups. The sub-group analysis was conducted according to the number of comorbidities and age.

**Results:**

Seven articles were selected in total for the meta-analysis. The mean Newcastle-Ottawa scale score was 8.14 points, suggesting studies were of high quality. There was a J-shaped dose-response relationship between alcohol consumption level and CVD incidence only in men. In general, light to moderate and moderate consumption lowered CVD incidence (Relative risk (RR) [95% confidence interval (CI)] was 0.68 [0.57–0.81] and 0.72 [0.58–0.90], respectively). In men with 3–4 comorbidities, there were no protective effects of light to moderate and moderate consumption on CVD incidence. In either groups of only men or men and women there were protective effects of light to moderate and moderate consumption on CVD incidence only in those aged between 41 and 65.

**Discussion:**

We found that light to moderate and moderate alcohol consumption had a protective effect on CVD incidence, there was no protective effect either in those with at least three comorbidities or people aged 40 or younger.

**Conclusions:**

We conclude that not all local community residents experience a protective effect of light to moderate consumption on CVD incidence. As such, it is necessary to recommend a moderate amount of drinking or less for each individual.

## Background

Alcohol consumption has existed throughout the majority of the history of mankind. However, making decisions about the consumption of alcohol remains difficult in consideration of the substantial body of literature examining its health benefits and risks [1]. Indeed, numerous studies have found various relationships (both positive and negative) between alcohol and several chronic and acute diseases. In particular, studies documenting the protective effects of alcohol on the incidence and mortality of cardiovascular diseases [1–7] and concerns about the impact of even small amounts of drinking on cancer incidence [1, 7, 8] are in direct conflict.

It has long been widely known and accepted that light to moderate drinking has a protective effect on cardiovascular diseases (CVD), such as coronary heart disease (CHD) and stroke. It has been reported that there is a J-or U-shaped relationship between the amount of drinking and CVD [9]. However, with the accumulation of new data, and discussions based on the analysis of this data, many studies are now being published opposing this traditional view point.

Previously, a meta-analysis was published investigating the relationship between alcohol consumption and coronary artery diseases between people who could not tolerate alcohol due to a genetic variance of an alcohol-degrading enzyme and people without this genetic variance [10]. The results of that study suggest that variations in alcohol dehydrogenase 1B (ADH1B) were associated with no or limited alcohol consumption, and the risk of CHD incidence was low in these subjects. This phenomenon was also seen in patients with light to moderate alcohol consumption. However, doubt remained in that study as to whether the cardio-protective effect of light to moderate drinking was a real effect. As a result, the authors suggested that there should be more studies investigating if the protective effects of alcohol were due to the influence of other factors. In contrast, the result of an additional meta-analysis containing 600,000 people found that drinking increased the risks of fatal myocardial infarction (MI), all kinds of stroke, fatal coronary diseases that were not MI, heart failure, and hypertensive diseases [11]. It revealed that it would be difficult to determine the amount of drinking that could lower the risks of CVD and argued that it could be necessary to lower the recommended dose of alcohol consumption in the existing guidelines. While not investigating CVD incidence directly, a recent meta-analysis examined the relationship of drinking and CHD mortality and it found there was no protective effect of light to moderate drinking in people under the age of 55 when heart health was controlled for in the analysis [12]. Finally, the results of an additional study also reported that there were no protective effects of light to moderate drinking on all-cause mortality in the elderly when the data were adjusted for exercise and perceived health status [13].

It is currently difficult to conduct a randomized controlled trial identifying any correlation between alcohol consumption and CVD incidence. As such, it is necessary to identify correlations through the results of prospective observational cohort studies. However, there are a number of studies with opposing data and view-points on this topic. This is a result of the challenges associated with effectively controlling confounding variables in observational studies. For example, specific and confounding demographics, including lifetime alcohol abstainers or former alcohol consumers who ceased consumption due to health problems, can reduce study accuracy or limit conclusions. Thus, it is reasonable to develop studies based on meta-analyses that select studies that only include appropriate and established standards. Particularly, in order to provide recommendation guidelines to the public, it is necessary to conduct meta-analyses of the studies performed in the community settings. However, it is difficult to find community-based studies conducted on the relationship between drinking and CVD incidence.

Therefore, the aim of this study was to investigate the dose-response relationship between alcohol consumption and CVD incidence through a meta-analysis of studies with people living in the local community. Further, we aimed to clarify whether light to moderate drinking has a protective effect on CVD incidence through a sub-group analysis.

## Methods

### Search strategy

We searched for relevant journals published prior to December 2017. Searches were made on PubMed, EMBASE, Cochrane Central Register of Controlled Trials (CENTRAL), PsycINFO, and Western Pacific Regional Index Medicus. To identify Korean journals, searches were made in Korean databases, including Research Information Sharing Service (RISS), KoreaMed, Korean Medical Database (KMbase), and National Digital Science Library (NDSL).

### Study selection criteria

Studies were selected using Co-evidence Software, the standard production platform of Cochrane Reviews (http://www.coevidence.org). Three authors (J.G.J., E.S.S., and J.E.J) excluded journals unsuitable for review based on the study titles and abstracts. If the titles and abstracts did not contain sufficient content for selection, their full texts were reviewed. The selection criteria are as follows: 1) prospective cohort studies and case-control studies analyzing the incidence of total CVD, all kinds of cardiovascular diseases, such as coronary heart disease (CHD) and stroke 2) alcohol consumption was the exposure condition, 3) the study reported at least three categories of alcohol consumption and a unit of alcohol measurement (g/day), 4) the outcome was development of total CVD, 5) a community or workplace setting, 6) subjects were initially free of CVD, 7) subjects were initially free of cancer and severe comorbidities which could trigger cardiovascular disease, such as uncontrolled hypertension, arrhythmia like atrial fibrillation, uncontrolled diabetes, and 8) the studies reported the outcome as either relative risks (RR), hazard ratios (HR), or odds ratios (OR) with 95% confidence intervals (CI). The exclusion criteria are as follows: 1) review articles, letters, conference abstracts, retrospective cohort studies, master’s or doctoral dissertations, and meta-analyses, 2) the studies did not report the incidence of total CVD as the outcome, 3) all animal studies, 4) any study with hospitalized populations, and 5) if the definition of a non-alcohol consuming subject was ambiguous (which did not distinguish social consumers and/or ex-consumers of alcohol).

### Definition of alcohol consumption categories

To examine the dose-response relationship, the alcohol consumption level was divided into six levels: Non-drinkers, light (0.01–10.0 g per day (g/day)), light to moderate (10.1–20.0 g/day), moderate (20.1–40.0 g/day), moderate to high (40.1–60.0 g/day), and high (> 60.0 g/day). Non-drinkers were used as a reference. Alcohol consumers were assigned to one of five alcohol groups according to the mid-point of the consumption ranges presented in each study. For example, if an alcohol consumer presented 23–46 g/day of alcohol, the individual was assigned to the moderate alcohol group (20.1–40.0 g/day), given the mid-point is 34.5 for this individual. If no upper limit of the consumption was presented in a study, it was set by adding 75% of the previous category to the lower limit. For example, if the maximum consumption category presented was > 46 g, and the previous category was 23 g, 63.25 g would be the upper limit value, since the sum of 46 and 75% of 23 is 63.25. An alcohol consumer who fell within that range was assigned to the moderate to high alcohol consumption group (40.1–60.0 g/day) given the mid-point of 46 and 63.25 is 54.63. Alcohol consumption was calculated from studies which were conducted based on the number of glasses by determining the standard glass size for the country in which the study was conducted and then using this to calculate the total g/day.

### Data extraction and quality assessment

Data were extracted from the studies selected by three authors (J.G.J., E.S.S., and J.E.J.), using a data collection form. To minimize issues related to the data collection form, a pilot test was conducted, and the data collection form was modified based on the findings from that study. Using this final form, the following information was extracted: the country in which the research was conducted, research setting, follow-up duration, age at baseline, the number of participants, the number of incident cases, the reference group data, comparison groups by level of alcohol consumption, research design, and controlled variables. The following information related to the meta-analysis was extracted: OR, HR, HR, 95% CI, the total number of participants, and the total number of events. The following information related to drinking-related information was extracted: measurement unit (g/day), criteria and/or classification of the level of alcohol consumption, and data collection method. Using the Newcastle-Ottawa Scale (NOS), the quality of the selected studies was evaluated [14]. The star-rating system of NOS consists of three categories: 1) selection (0–4 points); 2) comparability (0–3 points); and 3) outcome (0–3 points). A study was determined to of high quality if it obtained score higher than seven out of nine points.

### Statistical analysis

The effect size was expressed as an RR with 95% CI. The pooled RR estimates were calculated for each alcohol category. To normalize the data, we took the natural logarithms of the RR estimates for each level of alcohol consumption from the individual studies and used the natural logarithms of the 95% CI reported to calculate the standard errors of the log RR estimates. If there were missing data, we contacted the corresponding author to acquire the appropriate data for the meta-analysis. We input the missing statics, e.g. 95% CIs, after carefully determining the statistics (*p*-value) that allowed the calculation. In order to present more conservative analysis than the fixed-effect model, all pooled outcomes were determined applying a random-effect model, as suggested by DerSimonian and Laird [15].

The heterogeneity among the studies was estimated by Cochran Q test (*p* < 0.1 was set to be statistically significant heterogeneity) and the I^2^ statistic [16]. We considered studies with an I^2^ > 60% to contain substantial heterogeneity. To identify cases in which alcohol consumption had a protective effect on CVD incidence, a sub-group analysis was conducted according to the number of comorbidities (0, 1–2, and 3–4) and age (40 or younger, 41–65, over 65, and all those aged 20 or more). Publication bias was visually assessed based on the asymmetry of the funnel plots [17]. All meta-analyses were conducted using RevMan Software, Version 5.3 (Copenhagen; The Nordic Cochrane Center, The Cochrane Collaboration, 2014).

## Results

### Study characteristics

**Fig. 1:**
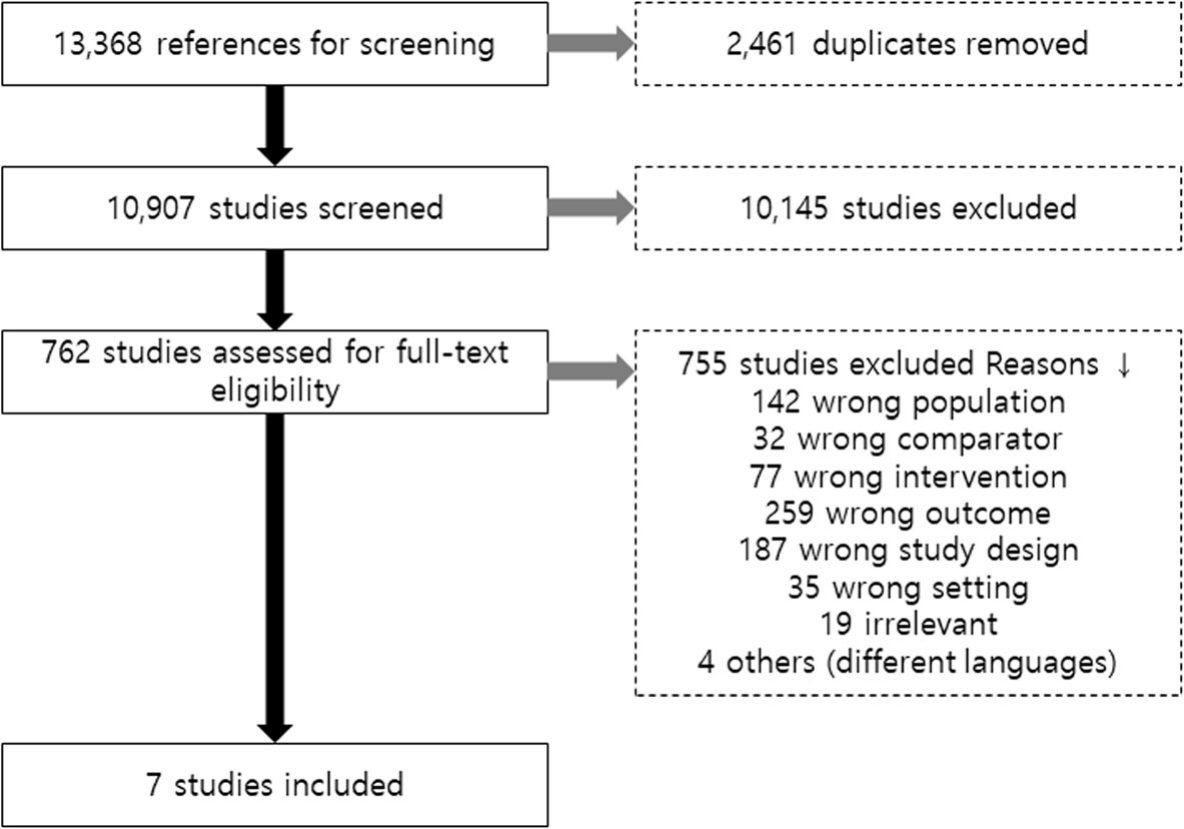
PRISMA chart

Of a total of 13,368 journal articles identified, 10,907 articles were selected through their titles and abstracts. After the exclusion of 2461 duplicates, 762 articles were determined to be suitable for full-text review. Of these, a total of seven articles met the criteria for selection and exclusion. The flow chart of the selection of studies is shown in Fig. 1. The characteristics of the journals included are stated in Table 1. Follow-up duration ranged from 4 to 13 years. All seven journal articles were prospective cohort research studies. All of the studies included were from high quality journals, and the mean NOS score was 8.14 (ranged from 7 to 9).

**Table 1:** Characteristics of the included studies

Author Year	Country	Setting	Follow-up (years)	Age range (baseline)	No. of total participants	No. of total incident cases	Reference group	Comparison groups (level of alcohol intake)	NOS score (1–9)	Study design
Burke V, 2007^21^	Australia	Community	11.6	15–88	258 (men) 256 (women)	44.57% (men) 46.48% (women)	Lifetime abstainer	≤40 g/day, 41-60 g/day, 61-120 g/day, 120-150 g/day, > 150 g/day (men) ≤20 g/day, 21-40 g/day, 41-80 g/day, 81-120 g/day, > 120 g/day (women)	7	Cohort
Higashiyama A, 2013^22^	Japan	Community	13.0	30–79	2336 (men)	9.25%	Never drinkers	≤23 g/day, 23-46 g/day, > 46 g/day	9	Cohort
Ikehara S, 2009^23^	Japan	Community	9.9	40–69	19,356 (men)	4.32%	Never drinkers	0.1–21.3 g/day, 21.4 g–42.7 g/day, 42.8 g–64.1 g/day, ≥64.2 g/day	9	Cohort
King DE, 2008^24^	USA	Community	4	51–70	7359 (men+women)	1.06%	Non drinkers	≤2 drinks/day, > 2drinks/day (men) ≤1 drink/day, > 1 drink/day (women)	8	Cohort
Iso H, 1995^20^	Japan	Community	10.5	40–69	2890 (men)	7.79%	Never drinkers	1-20 g/day, 21-41 g/day, 42-69 g/day, ≥70 g/day	9	Cohort
Snow WM, 2009^18^	Canada	Community	10	18–64	580 (men) 574 (women)	36.03% (men) 30.49% (women)	Never and occasional (< 0.65 g/day) drinkers	0.65–5.77 g/day, 5.78–18.1 g/day, > 18.1 g/day (men) 0.65–2.92 g/day, 2.93–9.15 g/day, > 9.15 g/day (women)	7	Cohort
Smyth A, 2015^19^	12 countries	Community	4.3 (3.0–6.0)	35–70	114,970 (men+women)	2.38%	Never drinkers	< 1 drink/day, 1-3drinks/day, >3drinsk/day (men) < 1 drink/day, 1–2 drinks/day, > 2 drinks/day (women)	8	Cohort

*NOS*: Newcastle-Ottawa scale

In this study, total CVD was defined as coronary heart disease and stroke (ischemic, hemorrhagic) in all selected articles. However, Snow et al. [18] included also other kinds of CVD (valvular heart diseases, heart failure, aortic diseases, stroke, peripheral artery diseases, etc.) besides coronary heart disease. Smyth A et al. [19] covered admission to hospital for heart failure as well as CVD. Hypertension, diabetes and dyslipidemia were included in all studies could be identified comorbidities. Besides, Iso H et al. [20] included hypertensive ophthalmoscopic change and left ventricular hypertrophy. In addition, using anti-thrombolytics was defined as one of comorbidities in a study by Smyth A et al. [19].

Age, sex, BMI, smoking, hypertension or blood pressure, diabetes, dyslipidemia or cholesterol level were adjusted as confounding factors in all selected studies. Besides, socio-demographic factors such as marital status, education level were also adjusted in some studies.

### Dose-response relationship between alcohol consumption and CVD incidence by sex

**Fig. 2:**
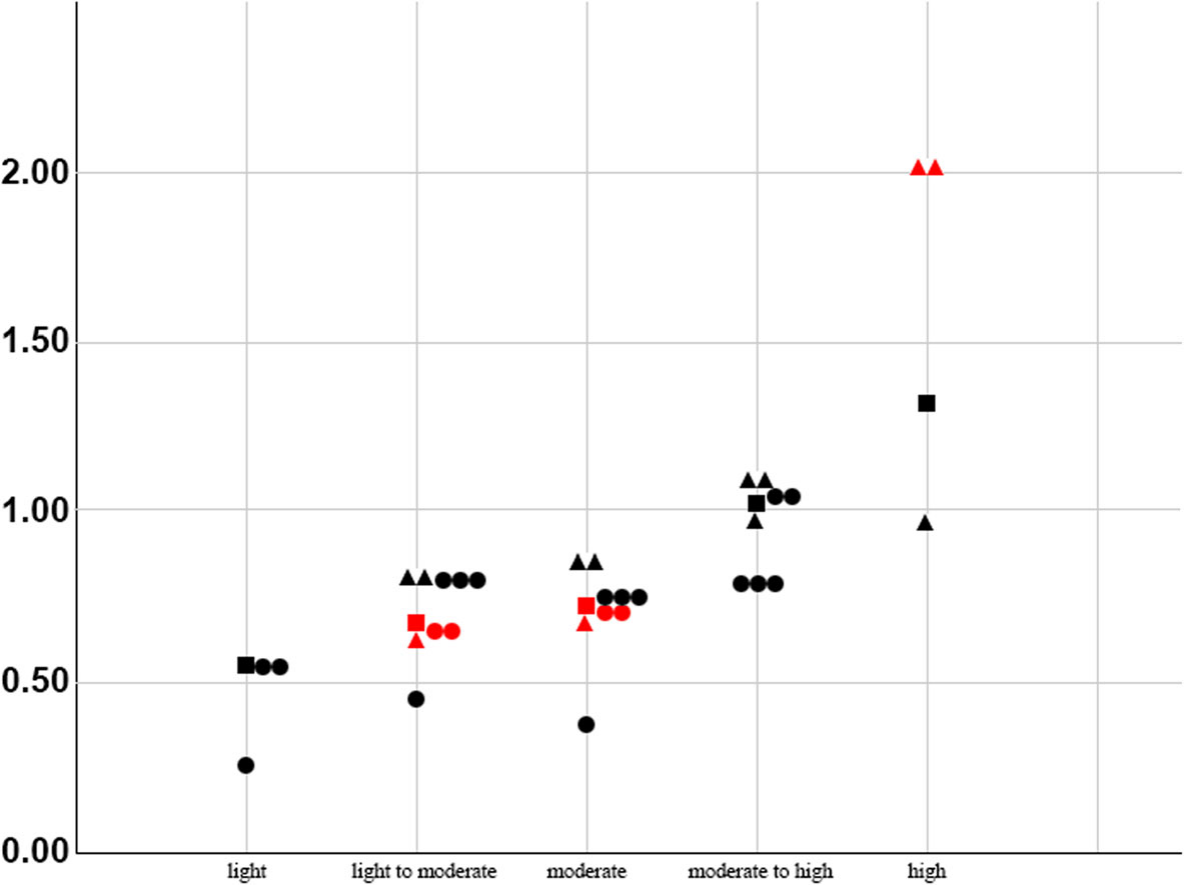
The relative risks of the incidences of cardiovascular diseases according to level of alcohol consumption when compared with non-drinkers in men only. Square: total of relevant studies; 1 triangle: comorbidity 1–2; 2 triangles: comorbidity 3–4; 1 circle: age ≤ 40; 2 circles: age 41–65; 3 circles: age ≥ 20. Red-colored means statistically significant

We found a J-shaped association between alcohol intake and CVD incidence in men. However, we were unable to identify the set point for the amount of drinking at which CVD incidence begins to increase. Further, we found that light to moderate and moderate alcohol consumption both lowered CVD incidence rates (RR [95% CI] was 0.68 [0.57–0.81] and 0.72 [0.58–0.90], respectively) (Table 2, Fig. 2).

**Table 2:** The relative risks of the incidences of cardiovascular diseases according to level of alcohol consumption when compared with non-drinkers

	Light (0.01-10 g/day)			Light to moderate (10.1-20 g/day)			Moderate (20.1-40 g/day)			Moderate to high (40.1-60 g/day)			High (60.1-120 g/day)		
	N	I^2^	RR (95% CI)	N	I^2^	RR (95% CI)	N	I^2^	RR (95% CI)	N	I^2^	RR (95% CI)	N	I^2^	RR (95% CI)
Men															
Total	1	–	0.54 (0.28–1.04)	4	0	0.68 (0.57–0.81)	4	16	0.72 (0.58–0.90)	3	0	1.02 (0.84–1.23)	2	89	1.32 (0.61–2.86)
Comorbidity = 0	–	–	–	1	–	0.54 (0.27–1.08)	1	–	0.40 (0.19–0.84)	–	–	–	–	–	–
Comorbidity = 1–2	–	–	–	1	–	0.64 (0.51–0.80)	1	–	0.71 (0.57–0.88)	1	–	0.99 (0.79–1.24)	1	–	0.91 (0.69–1.20)
Comorbidity = 3–4	–	–	–	2	0	0.80 (0.58–1.11)	2	0	0.85 (0.61–1.18)	2	0	1.09 (0.76–1.56)	1	–	2.00 (1.30–3.08)
Age ≤ 40	1	–	0.26 (0.05–1.35)	1	–	0.45 (0.08–2.53)	1	–	0.37 (0.06–2.28)				–	–	–
Age 41–65	1	–	0.54 (0.28–1.04)	3	0	0.66 (0.54–0.80)	3	44	0.71 (0.52–0.96)	2	0	1.04 (0.85–1.26)	–	–	–
Age ≤ 65	–	–	–	–	–	–	–	–	–	–	–	–	–	–	–
Age ≤ 20	–	–	–	1	–	0.80 (0.51–1.25)	1	–	0.74 (0.41–1.34)	1	–	0.79 (0.37–1.69)	–	–	–
Women															
Total	1	–	0.67 (0.31–1.45)	1	–	0.73 (0.34–1.57)	–	–	–	–	–	–	–	–	–
Comorbidity = 0	–	–	–	–	–	–	–	–	–	–	–	–	–	–	–
Comorbidity = 1–2	–	–	–	–	–	–	–	–	–	–	–	–	–	–	–
Comorbidity = 3–4	–	–	–	–	–	–	–	–	–	–	–	–	–	–	–
Age ≤ 40	1	–	**0.14 (0.05–0.39)**	1	–	0.50 (0.20–1.25)	–	–	–	–	–	–	–	–	–
Age 41–65	1	–	0.67 (0.31–1.45)	1	–	0.73 (0.34–1.57)	–	–	–	–	–	–	–	–	–
Age ≥ 65	–	–	–	–	–	–	–	–	–	–	–	–	–	–	–
Age ≤ 20	–	–	–	–	–	–	–	–	–	–	–	–	–	–	–
Men + Women															
Total	1	–	0.97 (0.87–1.08)	2	0	0.94 (0.76–1.17)	3	50	0.86 (0.61–1.22)	1	–	1.16 (0.66–2.04)	2	0	0.94 (0.44–2.00)
Comorbidity = 0	–	–	–	1	–	1.47 (0.59–3.66)	2	67	0.88 (0.38–2.01)	–	–	–	–	–	–
Comorbidity = 1–2	–	–	–	–	–	–	–	–	–	–	–	–	–	–	–
Comorbidity = 3–4	–	–	–	1	–	0.92 (0.74–1.14)	1	–	0.92 (0.76–1.11)	–	–	–	–	–	–
Age ≤ 40	–	–	–	–	–	–	–	–	–	–	–	–	–	–	–
Age 41–65	–	–	–	–	–	–	1	–	**0.62 (0.40–0.96)**	–	–	–	1	–	1.42 (0.41–4.92)
Age ≤ 65	–	–	–	–	–	–	–	–	–	–	–	–	–	–	–
Age ≤ 20	–	–	–	–	–	–	2	7	0.96 (0.74–1.23)	–	–	–	1	–	0.73 (0.28–1.90)

‘-‘means non-applicable

*N*: Number of included studies; *RR*: Relative risk; *CI*: Confidence interval

There were not a sufficient number of studies to identify or determine a dose-response relationship in women (Table 2). In the analysis of studies including both men and women, there was no clear dose-response relationship, and we were unable to confirm the amount of alcohol consumption that decreases or increases CVD incidence rate (Table 2).

### The protective effect of light, light to moderate, and moderate consumption on CVD incidence: sub-group analysis according to comorbidities and age

Light to moderate consumption lowered CVD incidence in men with 1–2 comorbidities (RR [95% CI] was 0.64 [0.51–0.80]). However, in those without comorbidities or those with 3–4 comorbidities, it did not lower CVD incidence (RR [95% CI] was 0.54 [0.27–1.08] and 0.80 [0.58–1.11], respectively). Moderate consumption lowered CVD incidence in those without comorbidities and with 1–2 comorbidities (RR [95% CI] was 0.40 [0.19–0.84] and 0.71 [0.57–0.88], respectively); however, in those with 3–4 comorbidities, moderate consumption did not lower CVD incidence (RR [95% CI] was 0.85 [0.61–1.18], Table 2, Fig. 2).

In men aged between 41 and 65, both light to moderate and moderate consumption lowered CVD incidence (RR [95% CI] was 0.66 [0.54–0.80] and 0.71 [0.52–0.96], respectively). However, in those aged 40 or younger and all those aged 20 or more, it did not lower CVD incidence (Table 2, Fig. 2).

In women, only light consumption lowered CVD incidence in those aged no more than 40 years (RR [95% CI] was 0.14 [0.05–0.39], Table 2).

In the analysis of studies including both men and women, there was no amount of consumption that lowered CVD incidence, even if values were divided by the number of comorbidities. In those aged between 41 and 65, only moderate consumption lowered CVD incidence (RR [95% CI] was 0.62 [0.40–0.96]), and when individuals aged 20 or older were included, it did not lower CVD incidence (Table 2).

### Harmful effects of high and very high drinking on CVD incidence

In either groups with men only or both men and women included, high consumption levels did not increase CVD incidence. However, in a sub-group analysis, high consumption levels increased CVD incidence in men with 3–4 comorbidities (RR [95% CI] was 2.00 [1.30–3.06], Table 2).

### Heterogeneity and sub-group analysis

There was heterogeneity in two studies that were only included in the case of high consumption in men (I^2^ = 89%), with no significant heterogeneity in all the other analyses (Table 2). There was a dose-response relationship, which was J–shaped, only in men. There was a similar J-shaped association in the results from a sub-group analysis according to comorbidities and age. However, the J-shape was not distinctive in some cases (P of comorbidity and age for interaction > 0.01, Fig. 2).

### Publication bias

We did not detect any significant publication bias in the visual inspection of the funnel plots (Additional file 1 Figure S1).

## Discussion

In the current study, we found that in men there was a dose-response J-shaped relationship between alcohol consumption and CVD incidence. However, if women were included in the analysis, no distinctive dose-response relationship was established. While we found that light to moderate and moderate alcohol consumption had a protective effect on CVD incidence, there was no protective effect either in those with at least three comorbidities or people aged 40 or younger. As such, our study and analyses were unable to establish criteria for the amount of consumption that could increase CVD incidence in people living in local communities. However, we did find that CVD incidence increased in those with three or more comorbidities who consumed more than 60 g/day.

Our results showing that light to moderate consumption had a protective effect on CVD incidence and that there was a J-shaped dose-response relationship in men are in line with the results of the previous studies [3–6, 9]. Light to moderate consumption lowers CVD incidence due to a lower CHD incidence rate, including MI, and because it reduces the progression of atherosclerosis by increasing high-density lipoprotein (HDL) cholesterol and lowering blood coagulation factors [5, 21]. Although not included in the meta-analyses of this study, other cohort studies which examined alcohol consumption found that the occurrence of MI or CHD in healthy, typical subjects also showed similar results [22, 23].

Recently, it has been reported by several studies that light to moderate alcohol consumption does not have a protective effect against stroke, among cardiovascular diseases except for CHD [6, 11]. Since our study analyzed CVDs as a whole, it is hard to separate and relate our findings only to CHD and stroke. However, it is possible to explain the relationship of light to moderate consumption and stroke, as our study showed that light to moderate drinking did not have a protective effect on CVD incidence in people with at least three comorbidities, and high consumption levels increased the risk of CVD development. The comorbidities investigated in this study included the diseases that may affect the occurrence of CVD, such as hypertension, diabetes mellitus, and dyslipidemia. Since alcohol increase the incidence of atrial fibrillation and can increase blood pressure, it could be logical that it may also increase the risk of stroke, which does not only occur due to atherosclerosis. While alcohol does have positive effects on atherosclerosis, such as increasing HDL cholesterol, the increased stroke incidence in those with several or a multitude of comorbidities may affect overall CVD incidence. Thus, we conclude it is possible that the protective effects on overall CVD incidence are outweighed by the negative impacts. The results of our study suggest there was a protective effect of light to moderate alcohol consumption on CVD incidence only in men aged between 41 and 65, and this was not apparent in analyses including young people aged 40 or younger. As discussed in past studies [12, 24], many elderly become non-consumers as a result of health problems that have developed with age. This may result in cardio-protective effects of light to moderate alcohol consumption when compared to elderly non-consumers.

In this study, unlike in men-only cohorts, it was difficult to evaluate any clear dose-response relationships either in women only or studies with both men and women groups. We suggest that the effect of consumption was difficult to discern due to an insufficient number of women for the meta–analysis. This is likely due to typical women living in the local communities’ rarely consuming more than moderate levels. Further, it could also be due to women having a lower risk of CVD incidence when compared to men.

In the J-shaped dose-response relationship between alcohol and CVD incidence in men in this study, the nadir point was 10.1-20 g/day. This was a lower value in comparison with that in previous studies [3, 4]. It seems this could be due to three out of the four studies in Japan that analyzed subjects with light to moderate consumption. There were insufficient studies to examine the regional differences in this study. However, the amount of consumption that may decrease CVD incidence is likely to be less in many Asians communities, given the alcohol-degrading enzyme is decreased physiologically in these individuals when compared to those of Western communities.

The limitations of this study are as follows. First, all the studies included were cohort research studies. Since they were observational studies, there is likelihood that not all confounding variables were controlled effectively. Second, it is likely that the actual amount of consumption could have been either overestimated or underestimated, as this study designated the alcohol groups at 20 g units, and consumers were assigned to each group based on the average of the categories presented in each case.

Despite these limitations, this study is significant in that it conducted a meta-analysis of high quality studies of individuals living in the local communities only. As a result of this study, a J-shaped dose-response relationship was observed between alcohol consumption and CVD incidence in men only. Further, we determined that light to moderate consumption lowered CVD incidence. However, since alcohol did not have a protective effect in all cases, we suggested that specific recommendation guidelines should be provided for alcohol consumption for each individual condition (e.g. age, sex, comorbidities, etc.).

## Conclusion

This study showed that light to moderate and moderate alcohol consumption had a protective effect on CVD incidence, there was no protective effect either in those with at least three comorbidities or people aged 40 or younger. We conclude that not all local community residents experience a protective effect of light to moderate consumption on CVD incidence. Therefore, it is necessary to recommend a moderate amount of drinking or less for each individual.

## Supplementary information

**Additional file 1: Figure S1.** Funnel plot of light to moderate drinking in men. Funnel plot of moderate drinking in men

## Data Availability

Not applicable.

## Abbreviations

ADH: Alcohol Dehydrogenase
CHD: Coronary Heart Disease (CHD)
CVD: Cardiovascular Disease
MI: Myocardial Infarction
