# Supplementary material for: The protective effect of alcohol consumption on the incidence of cardiovascular diseases: is it real? A systematic review and meta-analysis of studies conducted in community settings
Source: BMC Public Health. 2020 Jan 21;20:90. doi: 10.1186/s12889-019-7820-z (PMC6971904; doi:10.1186/s12889-019-7820-z)

Additional file 1: Figure S1

Funnel plot of light to moderate drinking in men


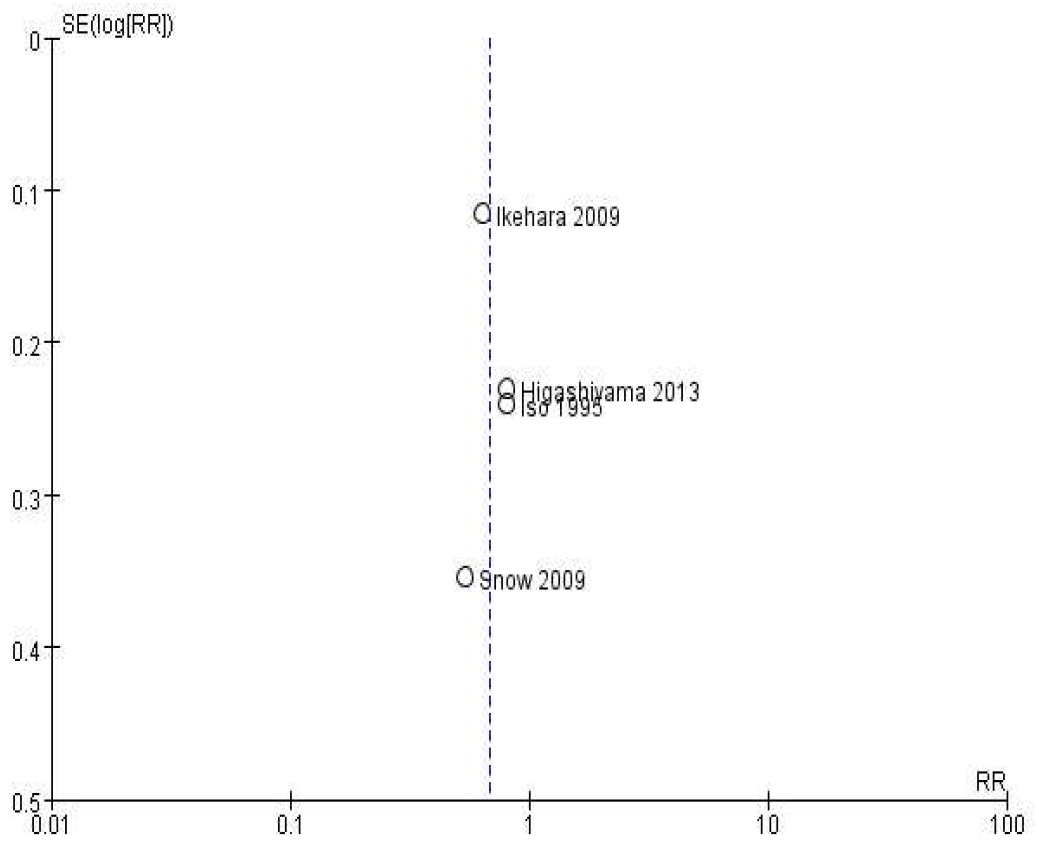


Funnel plot of moderate drinking in men


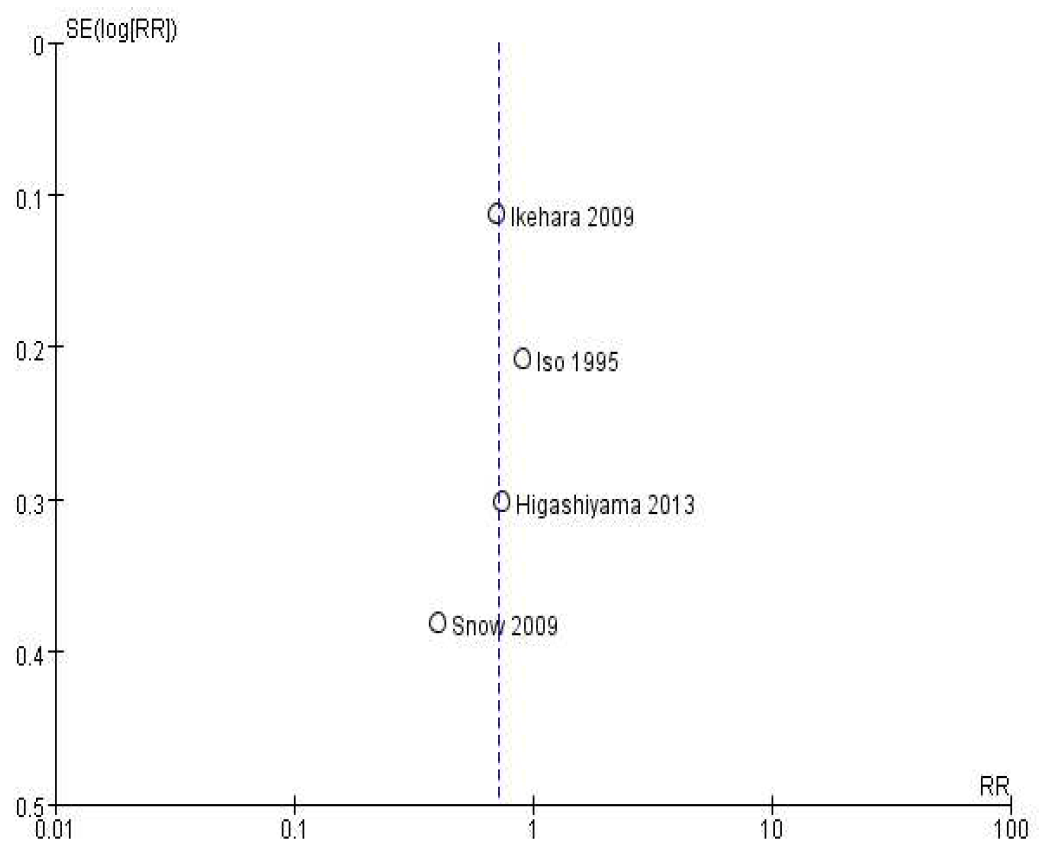

Supplement: Supplementary file 1 — Additional file 1: Figure S1. Funnel plot of light to moderate drinking in men. Funnel plot of moderate drinking in men [file 12889_2019_7820_MOESM1_ESM.docx]
